# Donor- and unit-specific factors influencing hemolysis in stored canine and feline packed red blood cells

**DOI:** 10.3389/fvets.2026.1816048

**Published:** 2026-06-12

**Authors:** Pablo Reguera, Carlos Martínez, Andreia Magalhães, Ana Queiros, Paula Barbosa, Ignacio Mesa

**Affiliations:** 1 Department of Internal Medicine, Aúna Especialidades Veterinarias IVC Evidensia, Valencia, Spain; 2 Banco de Sangue Animal, Porto, Portugal; 3 Animal Blood Bank, Barcelona, Spain

**Keywords:** canine, feline, hemolysis percentage, packed red blood cells, storage lesions, transfusion medicine

## Abstract

**Introduction:**

Hemolysis during storage compromises the quality of canine and feline packed red blood cell (pRBC) units by shortening unit shelf-life, reducing oxygen-carrying capacity, increasing the risk of non-immune-mediated hemolytic transfusion reactions, and necessitating the discard of units when stock is limited.

**Methods:**

Donor- and unit-level determinants of percent hemolysis were investigated in a multicenter quality-control dataset comprising 5,583 canine and 3,176 feline pRBC units. Associations were assessed by univariable screening followed by multivariable statistical analysis.

**Results:**

In both species, multivariable analysis indicated higher percent hemolysis with longer storage duration, older donor age, and lower unit packed cell volume (PCV). Beyond these common patterns, dogs exhibited higher percentage hemolysis in DEA 1 positive units and larger unit volume was associated with lower percentage hemolysis. Overall, 28.3% of canine units and 6.5% of feline units exceeded 0.8% hemolysis, whereas 16.5 and 4.0%, respectively, exceeded 1%. Threshold exceedance increased markedly from 22–28 days of storage onward.

**Discussion:**

Based on one of the largest multicenter veterinary datasets to date, these findings reaffirm that storage hemolysis increases with storage duration and is further shaped by donor- and unit-level characteristics. These results may support more precise donor selection and unit release policies and reinforce hemolysis testing of units stored for ≥28 days.

## 1 Introduction

Transfusion medicine is a fundamental component of the treatment of many medical and surgical conditions in companion animals, including the management of symptomatic anemia secondary to hemorrhage (trauma or surgery), immune-mediated hemolysis, or chronic kidney disease. To ensure continuous availability of blood products, blood banks must process and supply increasing numbers of units and store them for longer periods. Packed red blood cells are a blood component comprising erythrocytes suspended in an anticoagulant-preservative solution with nutrient additives and a small residual plasma volume (1). The current standard for handling of canine and feline pRBCs includes a maximum refrigerated storage time of 42 days (1). During refrigerated storage, blood cells maintain their metabolic activity, undergoing slow detrimental changes within the cells and storage media (2, 3). These changes are collectively termed storage lesions (SLs) and can affect red blood cell (RBC) survival, function, and recipient response to transfusion (2, 3). Consequently, research into SLs is a growing focus in veterinary transfusion medicine.

Storage lesions arise through multiple mechanisms operating across different molecular levels and are commonly classified as biochemical and biomechanical (2, 3). These mechanisms include impaired RBC metabolism, accumulation of oxidative stress, an increased vesiculation rate, and accumulation of storage by-products (e.g., potassium, hydrogen ions, ammonia and proinflammatory cytokines) (2, 3). These changes in the blood unit lead to time-dependent molecular and morphological alterations that result in increased osmotic fragility of the cells and thereby increased susceptibility to *in vitro* and *in vivo* hemolysis (2, 3). Accordingly, the percentage hemolysis of a unit is widely used as a practical surrogate marker of SLs burden in transfusion medicine (4). In human medicine, a progressive increase in hemolysis percentage with storage duration is well established (5). Similar trends have been reported for canine and feline pRBC units, although the veterinary evidence base is comparatively limited, underscoring the need for species-specific data (6–12). Mechanical and environmental factors may also affect RBC viability, as hemolysis is highly influenced by the processing, storage, and administration protocols (9, 10).

Hemolysis is a limiting factor for the shelf life of stored pRBCs owing to its detrimental impact on oxygen transport and delivery to hypoxic tissues, as well as its potential to trigger non-immune-mediated hemolytic transfusion reactions (13). When free hemoglobin (HGB) exceeds plasma and cellular binding capacities, it becomes a significant vasoactive and redox-active protein, potentially toxic to the vascular, myocardial, and renal systems (14–23). Although these transfusion reactions are usually benign and require no treatment, fatal reactions in dogs have been described (24–26). Due to the reported harmful effects of free HGB, and because percentage hemolysis serves as a surrogate marker for other potentially damaging storage by-products, regulatory standards stipulate that human RBC products should have mean end-of-storage hemolysis less than 1% in the United States and less than 0.8% in Europe (i.e., in the United States this is a lot-level quality control criterion requiring, with 95% confidence, that ≥ 95% of units are < 1% at end of storage, whereas in Europe < 0.8% is applied per individual unit) (27, 28). While no studies have defined an exact acceptable level of hemolysis for canine and feline pRBC units, the Association of Veterinary Hematology and Transfusion Medicine (AVHTM) felt that following the human guidelines at 1% is safest (4).

The recent application of omics approaches in human transfusion medicine has reinforced the prior understanding of factors affecting SLs, highlighting the impact of donor characteristics such as genetic variables (e.g., glucose-6-phosphate dehydrogenase deficiency), donor gender, ethnicity and age, frequency of donation, and others (2, 29–66). These donor-related variables can increase RBC susceptibility to osmotic and oxidative hemolysis through differences in erythrocyte membrane composition, function and resilience, extracellular vesicles production, HGB levels and its stability, and antioxidant capacities (32, 33, 45, 67, 68). There are no previous veterinary investigations addressing the influence of these donor-specific factors on hemolysis in stored canine and feline pRBCs. In addition to donor-related biology, characteristics of the unit itself may further influence hemolysis during storage. In human stored pRBCs, total unit volume and PCV are associated with the extent of hemolysis (8, 10, 12, 69). By contrast, in dogs only a single investigation has assessed these unit-level factors and reported no association (8), and, to our knowledge, comparable evaluations are absent in cats. This represents a significant gap in the veterinary literature.

The objectives of the present study were: 1. to quantify the influence of donor- (sex, age, blood group and donation frequency) and unit-specific characteristics (total volume and PCV) on hemolysis in stored canine and feline pRBC units; 2. to characterize the effect of storage duration on pRBC hemolysis; and 3. to identify a maximum storage interval compatible with human blood-banking quality standards, and assess the need for quality-control testing before administering units stored for more than four weeks. We hypothesized that percentage hemolysis in stored canine and feline pRBCs would be affected by donor- and unit-specific characteristics, as well as by storage duration. We further anticipated that some units would exceed both the US FDA 1% hemolysis limit and the European 0.8% hemolysis threshold. Using harmonized procedures across three centers, this cross-species study assembles the largest quality-control dataset of canine and feline pRBC units to date, integrating donor, unit and storage-time information. This design is intended to generate practical evidence to support veterinary transfusion practice-enabling risk-based quality control of older units, more informed donor selection and unit triage, and inventory rotation policies that enhance patient safety while minimizing wastage.

## 2 Materials and methods

### 2.1 Case selection

Units collected between 1st January 2021 and 31st December 2023 at three Animal Blood Bank facilities in Spain, Portugal and the Benelux region (Banco de Sangre Animal, Barcelona, Spain/Banco de Sangue Animal, Porto, Portugal/Banque de Sang, Liège, Belgium) were eligible for study. Inclusion followed the centers established quality-management programme, under which every fifth consecutively processed pRBC unit was taken for routine quality testing. This systematic approach formed part of day-to-day operations and was not devised for this study, and no additional randomization or stratification was applied. These facilities belonged to the same company and were no affiliated with a single hospital. Instead, they distributed canine and feline pRBC units to multiple veterinary centers, most commonly referral hospitals, which maintained a limited local stock for continued storage and subsequent clinical use. All donors were healthy dogs and indoor cats included in a blood donor program, aged between 1 and 10 years, and weighing 20–60 Kg and 3.5–9 Kg, respectively. All dogs and cats had been vaccinated and dewormed. The canine donors also demonstrated negative infectious-disease screening results for the following agents: *Anaplasma* spp. (antibody testing), *Ehrlichia* spp. (antibody testing and PCR), *Babesia* spp. (PCR), *Leishmania* spp. (antibody testing and PCR), *Brucella* spp. (PCR), and *Dirofilaria immitis* (antibody and antigen testing). All feline donors were tested for Feline Immunodeficiency Virus (antibody testing), Feline Leukemia Virus (FeLV antigen testing and FeLV proviral testing), and *Mycoplasma haemofelis*, *Mycoplasma turicensis and Mycoplasma haemominutum* (PCR). Donor blood-group antigen typing was performed at recruitment using point-of-care immunochromatographic assays (Alvedia, Limonest, France). In dogs, the assay detects the canine erythrocyte DEA 1 antigen; results were recorded as DEA 1 antigen positive or negative (weak positives classified as positive per the manufacturer). In cats, AB-system antigens were typed and recorded as A, AB or B. Antigen-typing results were linked to each unit and included as categorical covariates in the analyses. Most complete blood count and biochemistry parameters were within the reference range, although occasional mild, clinically insignificant abnormalities were present and did not preclude donor eligibility or blood collection. Blood collections were held under the regular donation program from a blood bank. All venipunctures were performed by a specially trained veterinarian or veterinary nurse using low stress handling consistent with Fear Free Practice Certification. All data was obtained from routine procedures performed at the veterinary blood bank, and no unnecessary procedures were done to blood donors. Blood samples were collected after signed informed owner consent. The study complies with EU Directive 2010/63/EU and its national transpositions (Spain: RD 53/2013; Portugal: DL 113/2013; Belgium: Royal Decree of 29 May 2013). Ethical review and approval were not required for this study because it involved only *in vitro* analyses of pre-existing pRBC units prepared and stored by licensed veterinary blood banks in Spain, Portugal and Belgium; no additional animal procedures were performed specifically for the purposes of this research.

### 2.2 Collection and processing of the canine pRBCs units

Collection and processing were standardized across the three centers under a shared standard operating procedure. Dogs were placed in lateral recumbency without sedation, and the puncture area over a jugular vein was clipped of hair and aseptically prepared (chlorhexidine and alcohol). Jugular venipuncture was performed, and blood was allowed to flow by gravity into a commercial quadruple blood bag (Compoflex quadruple system, Fresenius Kabi, Germany), consisting of a primary bag for the collection of 450 ± 10 mL of blood with 63 mL of CPD anticoagulant (tri-sodium citrate, sodium phosphate and dextrose), an empty bag to collect the whole blood after leukocyte filtration, an empty bag for plasma storage and one bag with 100 mL of additive solution SAG-MAN containing adenine, dextrose, mannitol and sodium chloride. Approximately 11–13 mL/kg of blood was collected, and if an interruption in blood flow was noted, the needle was immediately repositioned. Collected whole blood units were immediately stored using refrigerated butanediol plates (Compocool, Fresenius Kabi, Germany), keeping a constant temperature of 22 ± 2 °C, and processed within 24 h. During this period, whole blood units underwent pre-storage leukoreduction using the integrated in-line leukoreduction filter (CompoFlow Select, Fresenius Kabi, Germany). Briefly, immediately before filtration, the primary whole blood bag was gently homogenized and suspended at least 1 m above the connected empty receiving bag, and the entire unit was filtered by gravity into that bag, which collected the leukoreduced whole blood, while the filter was maintained in a vertical position. Total filtration time was required to be longer than 5 min; if filtration exceeded 1 h, the blood was re-homogenized and filtration was continued under the same gravity conditions, and these units were subjected to mandatory quality control. At the end of filtration, air was returned through the bypass system to the primary bag, and the tubing below the filter was clamped and sealed before further component processing. Whole blood units were gently inverted and placed in the centrifuge (Megafuge 40R, Thermo Scientific, Waltham, MA, United States) cups eliminating void spaces by using manufactured plastic adaptors. Centrifuges underwent annual calibration and complied with audits required by the ISO 9001 quality-management system. Weight differences under 0.2 gr between opposite cups were tolerated. Units were centrifuged at 2000 × g for 15 min at 20 °C with 80 s of acceleration and 110 s of deceleration. Plasma was completely expressed into the transfer bag, using a manual plasma extractor (Terumo Medical Corporation, New Jersey, United States). A volume of 100 mL of SAG-MAN was added to the pRBC bag. Packed red blood cell units were stored at 4 °C in a dedicated refrigerator (Fiocchetti, Luzzara, Italy) until clinical use. During storage, units were gently turned twice weekly.

### 2.3 Collection and processing of the feline pRBCs units

Collection and processing were standardized across the three centers under a shared standard operating procedure. Whole blood units were collected using a specific feline semi-closed system without leukocyte depletion filters, consisting of a 50 mL syringe and a primary blood bag collection attached to the syringe with a sterile connector (CompoDock, Fresenius SE, Hesse, Germany). The collection system was sealed, sterilized with Ethylene Oxide, and 7 mL of CPD (tri-sodium citrate, sodium phosphate and dextrose) were added as anticoagulant to the syringe, under sterile conditions using a laminar flow hood (Cruma FL-1, Diantech Solutions S.L., Barcelona, Spain). After a complete physical examination, an intravenous catheter was placed on the cephalic vein, and mild sedation was applied intravenously using ketamine (4 mg/Kg), butorphanol (0.1 mg/Kg), and midazolam (0.2 mg/Kg); doses were titrated to effect and could vary between individuals. Once sedated, donors were restrained in sternal recumbency using a towel-wrap in a sphinx position, leaving only the head exposed and the airways unobstructed. The neck was dorsally extended and rotated laterally to expose the jugular vein, and a tourniquet was applied at the thoracic inlet to facilitate jugular engorgement. The puncture area over the jugular vein was then shaved and aseptically prepared using chlorhexidine and alcohol. Jugular venipuncture was performed, and blood was withdrawn applying negative pressure by gently pulling manually the syringe plunger. A maximum of 11 mL/kg was collected. During collection, the syringe was gently agitated to allow proper contact of the blood with the anticoagulant. The collected blood was then transferred to the blood bag through the sterile connection ensuring the maintenance of a closed environment. After that, the tubing was sealed (Composeal, Fresenius Kabi, Hesse, Germany), units were stored at room temperature (22 ± 2 °C) and processed within 24 h. The volume of pRBC units was calculated on the basis of their weight, considering that 1 mL of pRBC weights 1.085 g. Units were gently mixed and placed in the centrifuge cups (Megafuge 40R, Thermo Scientific, Massachusetts, United States) eliminating void space by using manufactured plastic adaptors. Centrifuges underwent annual calibration and complied with audits required by the ISO 9001 quality-management system. Weight differences under 0.2 g between opposite cups were tolerated. Whole blood units were centrifuged at 2000 g for 15 min at 20 °C (64.4 °F), with 80 s of acceleration and 110 s of deceleration. After centrifugation, plasma was withdrawn using a 30 mmL syringe through a 3-way valve. If it met the visual inspection criteria (volume greater than 25 mL and no signs of lipemia, hemolysis, or icterus), it was then transferred into a secondary transfer bag, and a sterile connection is made. Next, the volume of SAG-MAN (adenine, dextrose, mannitol and sodium chloride) was added according to the volume of the red blood cell concentrate, in a 1:2 ratio.

### 2.4 Evaluation of the canine and feline pRBCs units

For quality-control analysis, canine and feline pRBC units were gently mixed by inversion, and an aliquot (1.5 mL) was aseptically aspirated from the attached tubing using a sterile 5 mL syringe fitted with a 20G × 1 in (0.9 × 30 mm) needle under a laminar flow hood. Before sampling, the end of the tubing was disinfected with 70% alcohol and clamped; once the needle was in place, the tubing was unclamped to allow aspiration. After sampling, the tubing was heat-sealed proximal to the puncture site, allowing the punctured portion to be removed so that it did not remain as part of the final unit. The collected aliquot was then analyzed for PCV, total HGB, and supernatant HGB. Packed cell volume was obtained using a microhematocrit centrifuge according to standard methodology. Total HGB was measured using a specific analyzer (Hb 201 System, HemoCue Inc., Brea, CA.), according to the manufacturer’s protocol. After centrifugation (Centrifuge IEC Centra CL3R, Thermo Scientific.), the supernatant HGB was determined by spectrophotometry using an analyzer for low values of HGB (Plasma Low Hb, HemoCue Inc.), also according to the manufacturer’s protocol. Packed red blood cell units were tested for hemolysis either before their administration or at the end of the maximum storage period of 42 days, if not used. The percentage of hemolysis was obtained using the following formula (70):


$$\begin{array}{l} \%\text{hemolysis}=\text{supernatant}\;\mathrm{HGB}(g/\mathrm{dL})\\ \times (100-\mathrm{PCV})/\text{totalHGB}(g/\mathrm{dL}) \end{array}$$

### 2.5 Statistical analysis

For comparison by storage times, units were categorized in 7 groups: group 1 evaluated within 72 h after processing (t0); group 2 tested after 4–7 days of storage (t1); group 3 tested after 8–14 days of storage (t2); group 4 tested after 15–21 days of storage (t3); group 5 tested after 22–28 days of storage (t4); group 6 tested after 29–35 days of storage (t5); and group 7 tested after 36–42 days of storage (t6). To evaluate the influence of different parameters on the level of hemolysis after different storage times, the pRBCs units were also grouped according to donor blood type (dogs: DEA 1 positive and DEA 1 negative; cats: A, AB, and B), sex (male and female), age (1–10 years), number of donations, and by unit characteristics such as total volume (dogs: 90–150 mL, 151–200 mL, 201–250 mL, and 251–300 mL; cats: 15–20 mL, 21–25 mL, 26–30 mL, 31–35 mL) and PCV (dogs: <40%, 40–45%, 46–50%, 51–55%, 56–60%, 61–65%, 66–70%, 71–75%, 76–80, and >80%; cats: <40%, 40–45%, 46–50%, 51–55, >56%).

Continuous variables were summarized as means with standard deviations and, where appropriate, medians with interquartile ranges; categorical variables were reported as proportions with 95% confidence intervals. Associations with percentage hemolysis and PCV were examined using mixed-effects regression. Univariable models were fitted for each covariate; variables showing evidence of association were then entered into multivariable models to estimate independent effects. Prespecified interactions were assessed where relevant. Because a donor could contribute more than one unit, donor identity was included as a random intercept in all models; center was included to account for site-level differences. Overall effects were evaluated using Type II tests (Wald χ^2^ for generalized linear mixed models; F-tests for linear mixed models). For significant categorical predictors, estimated marginal means with Tukey-adjusted pairwise comparisons were obtained. Statistical significance was set at *α* = 0.05 (two-sided).

Percentage hemolysis is a strictly non-negative outcome for which right-skewness and a mean–variance relationship is plausible. A Gamma model with a log link was therefore pre-specified, as it accommodates these properties and yields multiplicative (relative) effect estimates that are readily interpretable. PCV was analyzed using Gaussian mixed models, appropriate for approximately symmetric continuous outcomes. Model adequacy was assessed using standard residual diagnostics for the chosen family and link.

Data from the three centers were harmonized prior to analysis (standardized variable names, units and category coding). We performed range and logic checks, resolved duplicates and obvious entry errors, and retained an auditable record of all cleaning steps. Analyses were conducted in R (v4.5.1) using scripted, version-controlled workflows. Mixed-effects models were fitted using the lme4 (v1.1–37) and lmerTest (v3.1–3) packages; estimated marginal means and pairwise comparisons were obtained with emmeans (v1.11.2); and Type II tests (Wald χ^2^ for generalized linear mixed models and F-tests for linear mixed models) were computed with the Anova function from the car package (v3.1–3). Package versions were recorded (e.g., openxlsx/haven for import; ggplot2/ggpubr and base graphics for figures).

## 3 Results

During the study period, 5,583 canine pRBC units were collected from 3,698 dogs. Of these, 3,107 (56%) were from males and 2,476 (44%) from females. DEA 1 status was 3,174 (57%) positive and 2,409 (43%) negative. Mean donor age was 4.79 ± 2.53 years (males 4.67 ± 2.50, females 4.95 ± 2.55). Mean unit volume was 186.66 ± 60.43 mL and mean PCV 60.83 ± 4.40%. Total HGB averaged 21.33 ± 4.17 g/dL and percentage hemolysis 0.61 ± 0.69%. Mean storage time was 21.20 ± 14.68 days. Hemolysis, PCV and total HGB values for canine units are summarized in Table 1. In total, 923 canine pRBC units (16.5%) surpassed 1% hemolysis (Table 2) and 1,580 (28.3%) exceeded 0.8% hemolysis during the study (Table 3).

**Table 1:** Hemolysis, packed cell volume (PCV), and total hemoglobin concentration (HGB) measured in stored canine packed red blood cells (pRBC) units for varying periods of time.

Storage time group	*n*	Hemolysis (%)		PCV (%)		Total HGB (g/dL)	
		Mean	SD	Mean	SD	Mean	SD
t0	1854	0.07	0.11	63	3	21.77	6.86
t1	47	0.31	0.70	61	4	21.45	1.60
t2	30	0.53	0.50	60	5	20.57	1.66
t3	70	0.76	0.70	60	5	20.73	2.02
t4	2073	0.89	0.75	61	6	21.01	1.65
t5	1,327	0.9	0.64	63	5	21.25	1.41
t6	182	0.86	0.56	64	6	21.45	1.30

Hemolysis in canine units increased progressively with storage duration. Values are mean and standard deviation (SD). PCV: packed cell volume; HGB: hemoglobin; *n*: number of units; t0: tested within 72 h after processing; t1: tested after 4–7 days of storage; t2: tested after 8–14 days of storage; t3: tested after 15–21 days of storage; t4: tested after 22–28 days of storage; t5: tested after 29–35 days of storage; t6: tested after 36–42 days of storage.

**Table 2:** Number of canine packed red blood cell (pRBC) units with hemolysis >1% (overall and by time point, t0–t6).

Storage time group	≤ 1% (*n* = 4,660)	> 1% (*n* = 923)
t0 (*n* = 1854)	99.84% (*n* = 1851)	0.16% (*n* = 3)
t1 (*n* = 47)	93.62% (*n* = 44)	6.38% (*n* = 3)
t2 (*n* = 30)	80.00% (*n* = 24)	20.00% (*n* = 6)
t3 (*n* = 70)	75.71% (*n* = 53)	24.29% (*n* = 17)
t4 (*n* = 2073)	74.53% (*n* = 1,545)	25.47% (*n* = 528)
t5 (*n* = 1,327)	74.91% (*n* = 994)	25.09% (*n* = 333)
t6 (*n* = 182)	81.87% (*n* = 149)	18.13% (*n* = 33)

*n*: number of units; t0: units tested within 72 h after processing; t1: units tested after 4–7 days of storage; t2: units tested after 8–14 days of storage; t3: units tested after 15–21 days of storage; t4: units tested after 22–28 days of storage; t5: units tested after 29–35 days of storage; t6: units tested after 36–42 days of storage; ≤ 1%: units not exceeding 1% of hemolysis; > 1%: units exceeding 1% of hemolysis.

**Table 3:** Number of canine packed red blood cell (pRBC) units with hemolysis >0.8% (overall and by time point, t0–t6).

Storage time group	≤ 0.8% (*n* = 4,003)	> 0.8% (*n* = 1,580)
t0 (*n* = 1854)	99.73% (*n* = 1849)	0.27% (*n* = 5)
t1 (*n* = 47)	93.62% (*n* = 44)	6.38% (*n* = 3)
t2 (*n* = 30)	80.00% (*n* = 24)	20.00% (*n* = 6)
t3 (*n* = 70)	65.71% (*n* = 46)	34.29% (*n* = 24)
t4 (*n* = 2073)	58.66% (*n* = 1,216)	41.34% (*n* = 857)
t5 (*n* = 1,327)	53.73% (*n* = 713)	46.27% (*n* = 614)
t6 (*n* = 182)	60.99% (*n* = 111)	39.01% (*n* = 71)

*n*: number of units; t0: units tested within 72 h after processing; t1: units tested after 4–7 days of storage; t2: units tested after 8–14 days of storage; t3: units tested after 15–21 days of storage; t4: units tested after 22–28 days of storage; t5: units tested after 29–35 days of storage; t6: units tested after 36–42 days of storage; ≤ 0.8%: units not exceeding 0.8% of hemolysis; > 0.8%: units exceeding 0.8% of hemolysis.

For feline donors, 3,176 pRBC units were collected from 2,264 cats. Of these, 1,744 (55%) were from females and 1,432 (45%) from males. Regarding blood type, 3,052 (96%) were type A, 81 (2.5%) were type B, and 43 (1.5%) were type AB. Mean donor age was 5.19 ± 2.43 years (males 5.07 ± 2.50, females 5.29 ± 2.37). Mean unit volume was 31.33 ± 2.83 mL and mean PCV 48.67 ± 3.15%. Total HGB averaged 16.86 ± 1.28 g/dL and percentage hemolysis 0.23 ± 0.40%. Mean storage time was 8.73 ± 12.82 days. Hemolysis, PCV and total HGB values for feline units are summarized in Table 4. In total, 126 feline pRBC units (4.0%) surpassed 1% hemolysis (Table 5) and 205 (6.5%) exceeded 0.8% hemolysis during the study (Table 6).

**Table 4:** Hemolysis, packed cell volume (PCV), and total hemoglobin concentration (HGB) measured in stored feline packed red blood cells (pRBC) units for varying periods of time.

Storage time group	*n*	Hemolysis (%)		PCV (%)		Total HGB (g/dL)	
		Mean	SD	Mean	SD	Mean	SD
t0	2,287	0.05	0.06	51	3	16.94	1.25
t1	52	0.11	0.16	52	3	16.68	1.02
t2	12	0.35	0.27	47	4	16.58	1.51
t3	49	0.48	0.21	48	5	16.66	1.13
t4	577	0.66	0.50	48	5	16.60	1.35
t5	183	0.89	0.57	48	5	16.70	1.35
t6	16	1.11	0.71	50	2	17.50	1.45

Hemolysis in feline units increased progressively with storage duration. Values are mean and standard deviation (SD). PCV: packed cell volume; HGB: hemoglobin; t0: tested within 72 h after processing; t1: tested after 4–7 days of storage; t2: tested after 8–14 days of storage; t3: tested after 15–21 days of storage; t4: tested after 22–28 days of storage; t5: tested after 29–35 days of storage; t6: tested after 36–42 days of storage.

**Table 5:** Number of feline packed red blood cell (pRBC) units with hemolysis >1% (overall and by time point, t0–t6).

Storage time group	≤ 1% (*n* = 3,050)	> 1% (*n* = 126)
t0 (*n* = 2,287)	99.96% (*n* = 2,286)	0.04% (*n* = 1)
t1 (*n* = 52)	100.00% (*n* = 52)	0.00% (*n* = 0)
t2 (*n* = 12)	91.67% (*n* = 11)	8.33% (*n* = 1)
t3 (*n* = 49)	97.96% (*n* = 48)	2.04% (*n* = 1)
t4 (*n* = 577)	88.91% (*n* = 513)	11.09% (*n* = 64)
t5 (*n* = 183)	72.13% (*n* = 132)	27.87% (*n* = 51)
t6 (*n* = 16)	50.00% (*n* = 8)	50.00% (*n* = 8)

t0: units tested within 72 h after processing; t1: units tested after 4–7 days of storage; t2: units tested after 8–14 days of storage; t3: units tested after 15–21 days of storage; t4: units tested after 22–28 days of storage; t5: units tested after 29–35 days of storage; t6: units tested after 36–42 days of storage; ≤ 1%: units not exceeding 1% of hemolysis; > 1%: units exceeding 1% of hemolysis; n: number of units.

**Table 6:** Number of feline packed red blood cell (pRBC) units with hemolysis >0.8% (overall and by time point, t0–t6).

Storage time group	≤ 0.8% (*n* = 2,971)	> 0.8% (*n* = 205)
t0 (*n* = 2,287)	99.87% (*n* = 2,284)	0.13% (*n* = 3)
t1 (*n* = 52)	98.08% (*n* = 51)	1.92% (*n* = 1)
t2 (*n* = 12)	91.67% (*n* = 11)	8.33% (*n* = 1)
t3 (*n* = 49)	93.88% (*n* = 46)	6.12% (*n* = 3)
t4 (*n* = 577)	81.80% (*n* = 472)	18.20% (*n* = 105)
t5 (*n* = 183)	55.74% (*n* = 102)	44.26% (*n* = 81)
t6 (*n* = 16)	31.25% (*n* = 5)	68.75% (*n* = 11)

t0: units tested within 72 h after processing; t1: units tested after 4–7 days of storage; t2: units tested after 8–14 days of storage; t3: units tested after 15–21 days of storage; t4: units tested after 22–28 days of storage; t5: units tested after 29–35 days of storage; t6: units tested after 36–42 days of storage; ≤ 0.8%: units not exceeding 0.8% of hemolysis; > 0.8%: units exceeding 0.8% of hemolysis; n: number of units.

In the univariable analysis, percentage hemolysis increased with longer storage time in both species (dogs: χ^2^(1) = 3829.86, *p* < 0.001; cats: χ^2^(1) = 12068.43, *p* < 0.001), was higher in lower PCV categories (dogs: χ^2^(7) = 417.34, *p* < 0.001; cats: χ^2^(3) = 531.10, *p* < 0.001), and showed an inverse relation with unit volume, such that smaller units had higher hemolysis (dogs: χ^2^(1) = 888.21, *p* < 0.001; cats: χ^2^(1) = 260.56, *p* < 0.001). In dogs, DEA 1 positive units exhibited higher hemolysis than DEA 1 negative (χ^2^(1) = 119.87, *p* < 0.001), whereas donor sex and number of prior donations showed no evidence of association (χ^2^(1) = 2.88, *p* = 0.090; χ^2^(1) = 0.51, *p* = 0.473). Given the extreme imbalance in feline AB blood group (>95% type A), this covariate was omitted from the regression models and is reported descriptively only. In cats, female donors had higher hemolysis than male donors (χ^2^(1) = 6.37, *p* = 0.012), and a greater number of prior donations was associated with lower hemolysis (χ^2^(1) = 25.91, *p* < 0.001). Tables A1 and A2 summarize the univariable model results for dogs and cats, respectively. To complement these model-based results, descriptive percentage hemolysis data and unit counts for the main grouped predictors are provided in the Supplementary material (Tables A3 and A4 for canine and feline pRBC units, respectively).

In the multivariable mixed-effects Gamma analysis (log link), several predictors remained associated with the percentage of hemolysis in both species. Longer storage was linked to higher hemolysis, rising by roughly 11% per week in dogs and by about 10% per week in cats (dogs: +11.18% per week [95% CI 11.12–11.24; +1.53% per day, 95% CI 1.52–1.53], *p* < 0.001; cats: +9.94% per week [95% CI 9.74–10.15; +1.36% per day, 95% CI 1.34–1.39], *p* < 0.001). PCV also persisted in both species, showing a non-linear inverse relation, with lower PCV bands associated with higher hemolysis (both *p* < 0.001).

**Figure 1:**
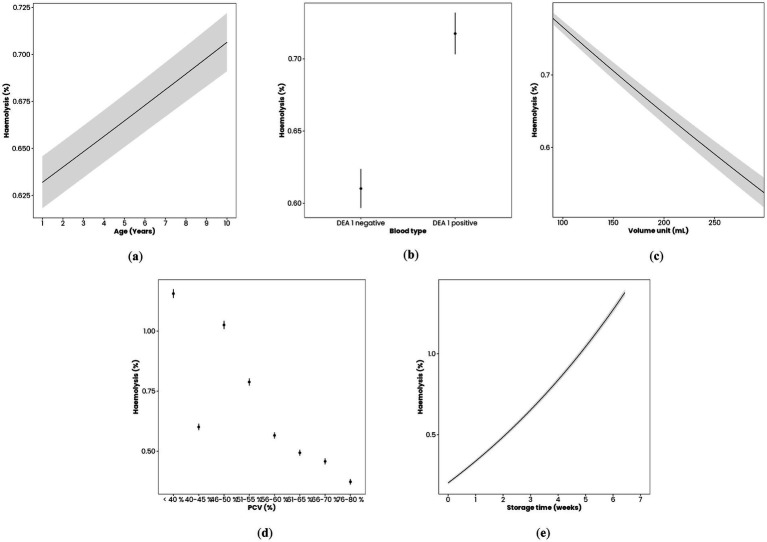
Model-adjusted percentage hemolysis in stored canine packed red blood cell (pRBC) units according to key predictors: **(a)** donor age; **(b)** blood type; **(c)** unit volume; **(d)** packed cell volume (PCV) category; and **(e)** Storage time. Values shown are adjusted estimates derived from the multivariable mixed-effects Gamma model and should not be interpreted as raw percentage hemolysis values from a single storage time point.

**Figure 2:**
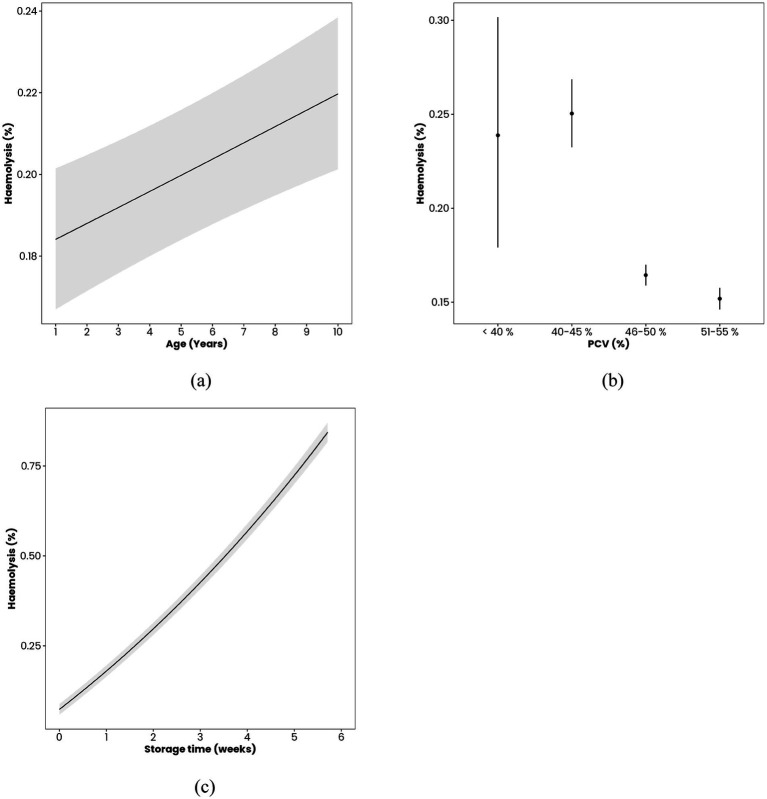
Model-adjusted percentage hemolysis in stored feline packed red blood cell (pRBC) units according to key predictors: **(a)** donor age; **(b)** packed cell volume (PCV) category; and **(c)** Storage time. Values shown are adjusted estimates derived from the multivariable mixed-effects Gamma model and should not be interpreted as raw percentage hemolysis values from a single storage time point.

Donor age remained positively associated, with each additional year corresponding to an approximately 0.50% relative increase in expected percentage hemolysis in dogs and a 0.33% relative increase in cats (dogs: +0.50% per year, 95% CI 0.44–0.55, *p* < 0.001; cats: +0.33% per year, 95% CI 0.18–0.48, *p* < 0.001), after adjustment for the other variables in the model. After adjustment, patterns differed between species. In dogs, DEA 1 positive units showed higher hemolysis than DEA 1 negative (about +6.6%, 95% CI 6.60–6.73, *p* < 0.001) and larger unit volume related to lower hemolysis (−0.69% per 10 mL, 95% CI − 0.74 to −0.65, *p* < 0.001). In cats, unit volume and number of prior donations were not retained (*p* = 0.070 and *p* = 0.714). Donor sex did not persist in either species, and number of prior donations did not persist in dogs either. Tables 7, 8 summarize the multivariable mixed-effects Gamma model results for dogs and cats, respectively. Figures 1a–e, 2a–c illustrate the adjusted associations from the multivariable mixed-effects Gamma models for dogs and cats, respectively. Because storage duration was included as a covariate in the multivariable models, these plots represent adjusted effects rather than raw percentage hemolysis values pooled across time points. To facilitate interpretation of the canine DEA 1 and unit-volume effects, the average storage age at testing for DEA 1 groups and unit-volume categories is provided in the Supplementary material (Table A5).

**Table 7:** Relative change in percentage hemolysis in dogs from a multivariable mixed-effects Gamma model.

Predictor	Contrast/scale	Relative change in hemolysis (%, 95% CI)	Test (stat)	*p*-value
Donor age	Per year	0.50% (0.44 to 0.55)	Wald t = 17.68	<0.001
Unit volume	Per 10 mL	−0.69% (−0.74 to −0.65)	Wald t = −29.75	<0.001
Storage duration	Per day	11.18% (11.12 to 11.24)	Wald t = 381.96	<0.001
Blood type (overall)	—	—	Type II χ^2^(1) = 41941.39	<0.001
PCV range (overall)	—	—	Type II χ^2^(7) = 6039583.13	<0.001

Estimates are multiplicative relative changes in percentage hemolysis from a multivariable mixed-effects Gamma model (log link) with a random intercept for donor ID. Continuous predictors are interpreted per unit as indicated (age per year; storage duration per day; unit volume per 10 mL). For multi-level predictors (blood type, PCV), the table shows overall Type II Wald χ^2^ tests; contrasts with the reference level are reported elsewhere. In dogs, longer storage is associated with higher hemolysis (≈11.18% per week; 95% CI 11.12 to 11.24). Hemolysis is higher in DEA 1-positive than in DEA 1-negative units. Across PCV bands, hemolysis is highest at <40% and lowest at 76–80%. PCV: packed cell volume; CI: confidence interval.

**Table 8:** Relative change in percentage hemolysis in cats from a multivariable mixed-effects Gamma model.

Predictor	Contrast/scale	Relative change in hemolysis (%, 95% CI)	Test (stat)	*p*-value
Donor age	Per year	0.33% (0.18 to 0.48)	Wald t = 4.29	<0.001
Storage duration	Per day	9.94% (9.74 to 10.15)	Wald t = 101.48	<0.001
PCV range (overall)	—	—	Type II χ^2^(3) = 114.81	<0.001

Estimates are multiplicative relative changes in percentage hemolysis from a multivariable mixed-effects Gamma model (log link) with a random intercept for donor ID. Continuous predictors are interpreted per unit as indicated (age per year; storage duration per day). For multi-level predictors (PCV), the table shows overall Type II Wald χ^2^ tests; contrasts with the reference level are reported elsewhere. In cats, longer storage is associated with higher hemolysis (≈9.94% per week; 95% CI 9.74 to 10.15). Hemolysis increases with donor age. Across PCV bands, hemolysis is highest at 40–45% and lowest at 51–55%. PCV: packed cell volume; CI: confidence interval.

## 4 Discussion

The present work represents the largest assessment of percentage hemolysis in stored canine and feline pRBC units. Furthermore, the present study was intended to address the influence of donor factors and unit characteristics in hemolysis. Consistent with previous veterinary and human work, hemolysis rose with increasing storage time (5). However, in contrast to the extensive human evidence base, veterinary studies demonstrating this rise are few and generally underpowered (6–12). Our findings therefore support this phenomenon in the largest cohort of canine and feline pRBC units reported to date.

At present, no veterinary study defines an exact, acceptable hemolysis threshold for stored canine and feline units. The Association of Veterinary Hematology and Transfusion Medicine recommends adopting the human standard and discarding units with >1% hemolysis at the end of storage or immediately before transfusion (4). Published canine and feline reports show that a proportion of units exceed 1% within the accepted shelf-life, most commonly beyond 28 days (8, 10). We observed the same distribution, with the great majority of >1% events clustering from day 28 onwards. In our cohort, if the US FDA human guideline had been applied to dogs, 333 of 1,327 units (25.09%) would have been discarded by week 5, whereas 33 of 182 units (18.13%) would have been discarded by week 6 (27). If the 0.8% European threshold had been applied to dogs, 614 of 1,327 units (46.27%) would have been discarded by week 5, whereas 71 of 182 units (39.01%) would have been discarded by week 6 (28). Likewise, if the 1% threshold had been applied to cats, 51 of 183 units (27.87%) would have been discarded by week 5 and 8 of 16 units (50%) by week 6. If the 0.8% European threshold had been applied to cats, 81 of 183 units (44.26%) would have been discarded by week 5 and 11 of 16 units (68.75%) by week 6. Considering these findings, percentage hemolysis should form part of routine quality control for stored pRBC units. The limited number of donors and units in many veterinary blood banks may, however, restrict the ability to discard non-conforming products, so when resources are tight a pragmatic interim approach is to prioritize testing of units approaching or exceeding 28 days of storage. The clinical significance of these findings should be interpreted with caution. In dogs, older pRBC units have been associated with increased odds of acute transfusion reactions, including febrile nonhemolytic and acute hemolytic reactions, which supports the clinical relevance of storage-related deterioration (24). However, the extent to which the relatively modest increases in percentage hemolysis observed in our study translate into clinically important recipient harm remains uncertain. Human data provide biological plausibility, because transfusion of older stored RBCs has been shown to increase post-transfusion hemolysis and circulating non-transferrin-bound iron, but randomized human studies have generally not demonstrated consistent worsening of major clinical outcomes with older standard-issue RBCs. Accordingly, our results support caution and targeted quality-control testing of older units, but they should not be interpreted as establishing a direct quantitative relationship between small increments in percentage hemolysis and adverse clinical outcomes.

Visual inspection of the pRBCs supernatant color is a simple alternative to evaluate hemolysis but is less accurate in both human and veterinary settings (71). In fact, even when the supernatant is red-tinged, this does not indicate that the 1% level of hemolysis has been surpassed (70). Thus, the hemolysis quantification based on cell-free hemoglobin measurement using portable devices is considered the reference method. Beyond identifying and excluding unsuitable units, quantitative hemolysis testing also prevents the unnecessary rejection of units that appear visually suboptimal for transfusion (70–72). Many veterinary centers keep pRBCs on site to ensure rapid access or to supply nearby practices without their own stock; however, portable analyzers are not routinely available in these settings, so once units leave the blood bank their percentage hemolysis usually cannot be verified locally.

In our series, mean PCV was broadly maintained during storage in both canine and feline units, with only a slight decline over time. This pattern differs from canine and human reports, where PCV tends to rise during storage. One possible explanation is that this change reflects water influx into erythrocytes as membranes are progressively altered, together with the osmotic effects of additive solutions (8, 69, 73–75). By contrast, our findings are consistent with the feline literature (10). Species differences may reflect physiological and metabolic particularities that drive distinct morphological responses during storage. One possibility is that feline erythrocytes, which are smaller and have a different surface-to-volume ratio, display lower osmotic fragility and a more limited capacity to swell, so PCV does not increase as observed in dogs and people (76).

Recognition of heterogeneity within RBCs has grown in recent years. Multiple studies show that pRBCs comprise subpopulations with distinct metabolic, physiological and biochemical profiles (47, 48, 77, 78). In human medicine, the final quality of pRBCs units correlates with donor factors-including age, sex, ethnicity, anemia and lifestyle-which together shape the biological age of the erythrocytes (32, 36, 42, 43, 47, 79–82). Unlike storage-induced aging, which is more extrinsic, each donor intrinsically contributes a unique spectrum of physical and metabolomic RBC profiles at the time of donation (47). Donor blood will naturally contain RBCs across a spectrum of biological ages, from young, newly matured cells to old, senescent ones. When a higher proportion of older erythrocytes is present, units show more hemolysis by-products, poorer post-storage recovery, reduced oxygen-delivery capacity and greater metabolic instability (13, 47, 53, 83, 84). This intrinsic, donor-dependent variability likely accounts for some of the differences in transfusion outcomes observed between ostensibly similar units (43, 81).

In our dataset, donor sex was not associated with percentage hemolysis in dogs. In cats, females showed a higher hemolysis percentage on univariable analysis, but this did not persist after multivariable adjustment. This contrasts with much of the human literature, where units from male donors typically exhibit greater hemolysis under osmotic, oxidative and mechanical stress and their erythrocytes are less deformable, findings attributed to androgen effects and to a larger proportion of biologically older erythrocytes, particularly in younger males (36, 43, 44, 47, 49–52, 81, 85). By comparison, female donors generally show more favorable storage profiles. Premenopausal status is associated with a younger circulating erythrocyte population, and estradiol and progesterone appear to enhance membrane deformability and reduce fragility, with these advantages waning after menopause as hormone levels fall (2, 36, 44, 47, 49–51, 85–87). Human studies also suggest that sex-mismatched transfusions may carry additional risk relative to sex-matched transfusions, potentially via antibody-mediated reactions, higher cell-free hemoglobin with increased nitric oxide scavenging, and differences in red-cell density and deformability that influence microvascular interactions (77, 88). Although the evidence is mixed and largely observational, it supports biological sex as a plausible modifier of transfusion outcomes in people. In dogs and cats, however, any sex-related effect may also be modulated by neuter status, since gonadectomy alters exposure to circulating sex hormones that are plausibly involved in red blood cell membrane stability, deformability, and oxidative susceptibility. Unfortunately, neuter status was not available in our dataset, so we could not assess whether it attenuated or obscured sex-related differences in hemolysis. This should be considered when interpreting our findings, especially because published data on spaying/neutering prevalence remain heterogeneous across populations, although neutering appears to be common in at least some companion-animal populations in southern Europe.

Across both species, greater donor age was associated with higher percentage hemolysis on univariable analysis, and this association remained after multivariable adjustment. Human data likewise point to a consistent role for age in cold storage hemolysis, measurable at roughly 0.04% points between younger and older donors (2, 36, 47, 51, 52). Red cells from older donors (roughly 50–75 years) exhibit more oxidative stress and less antioxidant capacity than those from younger adults (around 20–30 years), and age-related reductions in erythropoietin and hematopoietic stem-cell reserve may further compromise red-cell survival during storage. Recent work also shows that erythrocytes from young male donors (17–19 years) can display features of advanced biological age, including higher cell density, greater susceptibility to oxidative-stress hemolysis, and increased osmotic fragility (52). Taken together, the human literature suggests a non-linear, sex-modulated age effect, which is consistent with our finding that chronological age contributes to hemolysis risk while likely interacting with underlying red-cell biology.

Regarding blood group, dogs typed as DEA 1 positive had a higher percentage hemolysis than DEA 1 negative on univariable analysis, and the difference remained after multivariable adjustment. In cats, blood type was not assessed because the cohort was overwhelmingly type A, which precluded a meaningful comparison. Human studies have not shown an effect of ABO or other common blood-group systems on hemolysis during storage. Taken together, our findings point to a dog-specific association between blood type and percentage hemolysis, and further work is needed to clarify the mechanisms and clinical relevance.

Human studies indicate that donor ethnicity can influence hemolysis during storage, probably through differences in genetic background and the prevalence of hemoglobin variants (2, 32, 36, 37, 39, 44, 45, 48). Extending this question to veterinary donors is challenging because dog and cat populations are highly heterogeneous, with many breeds and crossbreeds and uneven representation across groups. Meaningful inference therefore requires large, multi-center datasets and breed-aware analyses, and at present robust conclusions about breed effects on in-bag hemolysis are not possible.

Concerning donation history, we found no association between the number of donations and percentage hemolysis in dogs. In cats, more frequent donors showed lower hemolysis on univariable analysis, but the effect disappeared after multivariable adjustment. Human data suggest only a modest and mixed influence of donation frequency on red-cell fragility (36, 45, 50, 53, 54). Frequent donation has been linked to reduced susceptibility to oxidative hemolysis, with little or no change in storage or osmotic hemolysis. These patterns may reflect iron depletion and lower ferritin, which are associated with lower in-bag hemolysis yet can impair post-transfusion recovery in experimental models.

Regarding unit characteristics, both species showed an influence on percentage hemolysis. On univariable analysis, higher PCV and greater unit volume were associated with lower hemolysis in dogs and cats. In multivariable models, dogs retained both effects, with the highest hemolysis at PCV < 40%, the lowest at 40–45%, and little additional change above 45%. In cats, only PCV remained independently associated, with higher hemolysis below 45% and clearly lower values at 46–55%, while volume no longer contributed. These results differ from a recent canine report that found no association and contrast with human data (8, 89), where higher PCV and larger volumes tend to show greater hemolysis, plausibly because denser and larger packs sustain more mechanical and oxidative stress and allow less efficient exchange of nutrients and gases during storage. In our dataset, the greater percentage hemolysis observed in lower-PCV and smaller-volume units most plausibly reflects process- and biology-related factors, particularly in dogs: prestorage leukoreduction through in-line filters and a fixed volume of additive solution increase shear exposure and the additive-to-red-cell ratio in smaller or more dilute concentrates, while small packs are more susceptible to thermal fluctuation and handling stress. In cats, units are prepared without leukoreduction and with additive titrated to the concentrate (≈1:2), which standardizes the additive-to-cell ratio and likely attenuates any independent volume effect; after adjustment, PCV remains the principal determinant. Species physiology may further contribute, as feline erythrocytes exhibit greater osmotic fragility and distinct antioxidant profiles compared with canine cells. Further work is needed to determine whether the effects of PCV and volume on hemolysis in dogs and cats genuinely diverge from those seen in human medicine.

In human transfusion medicine, the exposome—diet, medications, and environmental or occupational exposures—has been recognized as a modifier of red-cell storage biology and hemolysis (2, 9, 32, 33, 36, 56–66, 90). Omics studies show that non-genetic exposures imprint metabolic and redox phenotypes and change susceptibility to storage-, osmotic- and oxidant-stress hemolysis. By contrast, this area has not been systematically explored in veterinary transfusion medicine. Evidence in dogs and cats is essentially absent, so any effect of medications, nutrition or environment on in-bag hemolysis remains hypothetical and warrants dedicated investigation.

There are practical options to reduce red-cell storage lesions, and the strength of evidence differs between human and veterinary medicine. Pre-storage leukoreduction removes donor leucocytes and platelets before refrigeration, which limits cytokine release, microvesiculation and other mediator build-up during storage (91–93). In dogs this approach has been associated with more favorable in-bag and post-transfusion profiles and, more recently, with improved metabolic and redox omics signatures (91–93). Even so, the AVHTM TRACS consensus states that evidence in dogs and cats is still insufficient to recommend for or against routine leukoreduction. In human medicine, prestorage leukoreduction is associated with reduced febrile nonhemolytic transfusion reactions, but prospective canine studies have not demonstrated a comparable reduction in acute transfusion reactions or post-transfusion inflammatory biomarkers in dogs receiving leukoreduced compared with nonleukoreduced pRBCs (4). In our cohort, canine units were leukoreduced and feline units were not, which may contribute to species differences in hemolysis. Post-storage washing means rinsing stored red cells with isotonic solution immediately before use to remove supernatant cell-free hemoglobin, non-transferrin-bound iron, potassium, bioactive lipids and microvesicles. Human and translational studies indicate benefit when older units are washed, whereas washing very fresh units can increase circulating cell-free hemoglobin (94). Recent veterinary work in dogs shows that washing reduces supernatant injury mediators and improves selected in-vitro quality indices, though it causes red-cell loss and adds handling, so most authors view it as a selective option for older units or high-risk recipients (95). Rejuvenation seeks to restore the metabolic state of stored erythrocytes by replenishing ATP and 2,3-DPG, which can improve oxygen-unloading kinetics and deformability. Evidence is strongest in human studies and reviews, while veterinary data are preliminary. In dogs, an antioxidant-supplementation experiment using N-acetylcysteine, ascorbic acid and a vitamin-E analogue modulated oxidative markers but produced limited or inconsistent improvements in global storage-lesion endpoints, indicating that true rejuvenation protocols require dedicated veterinary trials before clinical adoption (50, 69).

The present study has several limitations. Firstly, its retrospective design meant that some donor variables were markedly unbalanced (e.g., feline blood group with >95% type A) or unavailable, most notably neuter status. Secondly, hemolysis measurements were performed only at unit issue or the end of storage; this prevents analysis of temporal hemolysis kinetics and may obscure early patterns. More intensive sampling was not pursued because reliability requires direct sampling from the unit—inspection of attached segments is not an accurate surrogate for in-bag changes—and each manipulation carries a contamination risk and causes cumulative volume loss. Thirdly, although collection and processing were standardized across the three centers under shared SOPs, small center-level micro-variations—such as brief storage-temperature fluctuations during transport or handling, differences in agitation/cooling rates, or other operational factors—may still influence red-cell fragility and hemolysis, potentially affecting viability despite accounting for center as a random effect in the models. Fourthly, the exact hold time from blood collection to processing was not recorded individually for each unit. Although all whole blood units were processed within a maximum of 24 h after collection according to the blood bank workflow, we could not assess whether variation in pre-processing hold time influenced percentage hemolysis. This is relevant because human transfusion-medicine studies suggest that overnight or delayed whole-blood processing may produce measurable *in vitro* changes in red blood cell quality, even when the resulting pRBC units remain within acceptable quality standards. Future studies should prospectively record the exact time from collection to processing for each unit in order to evaluate its potential effect more precisely. Finally, as an observational analysis of routine quality-control data, our study cannot establish causality, and unmeasured confounders may persist despite multivariable adjustment.

Taken together, these findings support a more individualized approach to quality assessment in stored canine and feline pRBC units. Storage hemolysis should not be regarded only as a time-dependent phenomenon, but also as an outcome influenced by donor- and unit-level characteristics. This may help inform more selective donor recruitment, more evidence-based decisions regarding the release of older units, and more targeted quality-control testing, particularly beyond 28 days of storage. Ultimately, applying this perspective in routine veterinary transfusion practice may contribute to improved patient safety while helping blood banks use limited blood-product inventories more efficiently, although the clinical impact of relatively small differences in percentage hemolysis remains to be defined more precisely in prospective recipient-based studies.

## Data Availability

The datasets presented in this article are not readily available because they are owned by Animal Blood Bank and are subject to confidentiality and data-sharing restrictions. Requests to access the datasets should be directed to the corresponding author and will be considered on a case-by-case basis, subject to approval by Animal Blood Bank.
