# Supplementary material for: Donor- and unit-specific factors influencing hemolysis in stored canine and feline packed red blood cells
Source: Front Vet Sci. 2026 Jun 12;13:1816048. doi: 10.3389/fvets.2026.1816048 (PMC13308521; doi:10.3389/fvets.2026.1816048)
Supplement: Supplementary file 1 [file Supplementary_file_1.pdf]

Donor- and unit-specific factors influencing hemolysis in stored canine and feline packed red blood cells

Appendix A

Appendix A.1

Table A1. Effects on percentage hemolysis in dogs: univariable Gamma (log-link) model

Predictor	df	χ^2	p-value
Storage duration (weeks)	1	3829.86	<0.001
PCV range (%)	7	417.34	<0.001
Unit volume (mL)	1	888.21	<0.001
Blood type	1	119.87	<0.001
Donor age (years)	1	7.61	0.006
Donor sex	1	2.88	0.090
Number of previous donations	1	0.51	0.473

Each row reports the Wald χ^2 test from a univariable Gamma model (log link). Predictor names include units where applicable (age in years; storage duration in weeks; unit volume in mL). PCV: packed cell volume; df: degrees of freedom

Appendix A.2

Table A2. Effects on percentage hemolysis in cats: univariable Gamma (log-link) model

Predictor	df	χ^2	p-value
Storage duration (weeks)	1	12068.43	<0.001
PCV range (%)	3	531.10	<0.001
Unit volume (mL)	1	260.56	<0.001
Donor age (years)	1	44.57	<0.001
Donor sex	1	6.37	0.012
Number of previous donations	1	25.91	<0.001

Each row reports the Wald χ^2 test from a univariable Gamma model (log link). Predictor names include units where applicable (age in years; storage duration in weeks; unit volume in mL). PCV: packed cell volume; df: degrees of freedom

This is a provisional file, not the final typeset article

Appendix A.3

Table A3. Descriptive distribution of grouped predictors and percentage hemolysis in canine packed red blood cell (pRBC) units.

**Predictor**	**Group**	**n**	**Percent hemolysis (%) Mean ± SD**
Donor sex	Male	3107	0.60 ± 0.66
	Female	2476	0.63 ± 0.73
DEA 1 status	DEA 1 negative	2409	0.49 ± 0.67
	DEA 1 positive	3174	0.70 ± 0.70
Final unit volume	90–150 mL	1814	0.94 ± 0.79
	151–200 mL	282	0.83 ± 0.75
	201–250 mL	2764	0.44 ± 0.56
	251–300 mL	723	0.34 ± 0.52
PCV category	<40%	12	2.19 ± 1.65
	40–45%	10	0.60 ± 0.41
	46–50%	56	1.49 ± 1.30
	51–55%	526	1.00 ± 0.93
	56–60%	2111	0.65 ± 0.70
	61–65%	2367	0.44 ± 0.56
	66–70%	454	0.70 ± 0.50
	71–75%	37	0.59 ± 0.37
	76–80%	9	0.62 ± 0.34

	>80%	1	2.32
Storage time	t0	1854	0.07 ± 0.11
	t1	47	0.31 ± 0.70
	t2	30	0.53 ± 0.50
	t3	70	0.76 ± 0.70
	t4	2073	0.89 ± 0.75
	t5	1327	0.90 ± 0.64
	t6	182	0.86 ± 0.56

Values are mean and standard deviation (SD). n: number of units; t0: tested within 72 h after processing; t1: tested after 4-7 days of storage; t2: tested after 8-14 days of storage; t3: tested after 15-21 days of storage; t4: tested after 22-28 days of storage; t5: tested after 29-35 days of storage; t6: tested after 36-42 days of storage.

This is a provisional file, not the final typeset article

Appendix A.4

Table A4. Descriptive distribution of grouped predictors and percentage hemolysis in feline packed red blood cell (pRBC) units.

**Predictor**	**Group**	**n**	**Percent hemolysis (%) Mean ± SD**
Donor sex	Male	1432	0.22 ± 0.40
	Female	1744	0.23 ± 0.39
Blood type	A	3052	0.21 ± 0.37
	AB	43	1.12 ± 0.98
	B	81	0.31 ± 0.39
Final unit volume	15–20 mL	15	0.84 ± 0.77
	21–25 mL	100	0.57 ± 0.62
	26–30 mL	927	0.34 ± 0.48
	31–35 mL	2134	0.16 ± 0.30
PCV category	<40%	14	0.85 ± 0.63
	40–45%	480	0.47 ± 0.52
	46–50%	1998	0.19 ± 0.37
	51–55%	659	0.13 ± 0.24
	>56%	25	0.51 ± 0.55
Storage time	t0	2287	0.05 ± 0.06
	t1	52	0.11 ± 0.16
	t2	12	0.35 ± 0.27

t3	49	0.48 ± 0.21
t4	577	0.66 ± 0.50
t5	183	0.89 ± 0.57
t6	16	1.11 ± 0.71

Values are mean and standard deviation (SD). n: number of units; t0: tested within 72 h after processing; t1: tested after 4-7 days of storage; t2: tested after 8-14 days of storage; t3: tested after 15-21 days of storage; t4: tested after 22-28 days of storage; t5: tested after 29-35 days of storage; t6: tested after 36-42 days of storage.

This is a provisional file, not the final typeset article

Appendix A.5

Table A5. Average storage age at testing for canine packed red blood cell (pRBC) units according to DEA 1 status and unit-volume category.

**Group**	**n**	**Mean storage age at testing (days) ± SD**
**A. DEA 1 status**		
DEA 1 negative	2409	18.74 ± 15.21
DEA 1 positive	3174	23.06 ± 13.99
**B. Unit volume**		
90–150 mL	1814	30.28 ± 7.02
151–200 mL	282	28.12 ± 10.51
201–250 mL	2764	16.90 ± 15.38
251–300 mL	723	12.17 ± 14.73

Values are presented as mean ± SD days from processing to hemolysis testing. n: number of units; SD: standard deviations
